# Phytochemical evaluation of *Ziziphus mucronata* and *Xysmalobium undulutum* towards the discovery and development of anti-malarial drugs

**DOI:** 10.1186/s12936-024-04976-1

**Published:** 2024-05-11

**Authors:** Muzi N. Buthelezi, Vhahangwele G. Tshililo, Abidemi P. Kappo, Mthokozisi B. C. Simelane

**Affiliations:** Department of Biochemistry, Faculty of Science, University of Johannesburg, Auckland Park Kingsway Campus, Johannesburg, South Africa

**Keywords:** *Plasmodium falciparum*, Malaria, *Ziziphus mucronata*, *Xysmalobium undulutum*, Betulinic acid, Lupeol, *Pf*Hsp70-1

## Abstract

**Background:**

The development of resistance by *Plasmodium falciparum* is a burdening hazard that continues to undermine the strides made to alleviate malaria. As such, there is an increasing need to find new alternative strategies. This study evaluated and validated 2 medicinal plants used in traditional medicine to treat malaria.

**Methods:**

Inspired by their ethnobotanical reputation of being effective against malaria, *Ziziphus mucronata* and *Xysmalobium undulutum* were collected and sequentially extracted using hexane (HEX), ethyl acetate (ETA), Dichloromethane (DCM) and methanol (MTL). The resulting crude extracts were screened for their anti-malarial and cytotoxic potential using the parasite lactate dehydrogenase (pLDH) and 3-(4,5-dimethylthiazol-2-yl)-2,5-diphenyltetrazolium bromide (MTT) assay, respectively. This was followed by isolating the active compounds from the DCM extract of *Z. mucronata* using silica gel chromatography and structural elucidation using spectroscopic techniques (NMR: ^1^H, ^12^C, and DEPT). The active compounds were then targeted against *P. falciparum* heat shock protein 70–1 (PfHsp70-1) using Autodock Vina, followed by in vitro validation assays using ultraviolet–visible (UV–VIS) spectroscopy and the malate dehydrogenase (MDH) chaperone activity assay.

**Results:**

The extracts except those of methanol displayed anti-malarial potential with varying IC_50_ values, Z. mucronata HEX (11.69 ± 3.84 µg/mL), ETA (7.25 ± 1.41 µg/mL), DCM (5.49 ± 0.03 µg/mL), and *X. undulutum* HEX (4.9 ± 0.037 µg/mL), ETA (17.46 ± 0.024 µg/mL) and DCM (19.27 ± 0.492 µg/mL). The extracts exhibited minimal cytotoxicity except for the ETA and DCM of *Z. mucronata* with CC_50_ values of 10.96 and 10.01 µg/mL, respectively. Isolation and structural characterization of the active compounds from the DCM extracts revealed that betulinic acid (19.95 ± 1.53 µg/mL) and lupeol (7.56 ± 2.03 µg/mL) were responsible for the anti-malarial activity and had no considerable cytotoxicity (CC_50_ > µg/mL). Molecular docking suggested strong binding between PfHsp70-1, betulinic acid (− 6.8 kcal/mol), and lupeol (− 6.9 kcal/mol). Meanwhile, the in vitro validation assays revealed the disruption of the protein structural elements and chaperone function.

**Conclusion:**

This study proves that *X undulutum* and *Z. mucronata* have anti-malarial potential and that betulinic acid and lupeol are responsible for the activity seen on *Z. mucronata*. They also make a case for guided purification of new phytochemicals in the other extracts and support the notion of considering medicinal plants to discover new anti-malarials.

**Supplementary Information:**

The online version contains supplementary material available at 10.1186/s12936-024-04976-1.

## Background

Malaria is a persistent killer disease caused by an apicomplexan protozoan parasite of the genus *Plasmodium*, with *Plasmodium falciparum* being the most lethal causative agent of malaria, accounting for over 99% of overall malarial deaths [1–3]. This disease poses a significant public health problem, especially in Africa and sub-Saharan Africa, as indicated by the 274 million clinical cases and 619 00 global deaths reported in the year 2021 [4]. Beyond this health toll, the repercussion of this disease extends into the socio-economic realm, where they act as commanding barriers that continue to stunt economic development, thus perpetuating the cycle of poverty [5]. Although strides have been made over the years by both governmental and non-governmental organizations to help curb the spread of malaria, the disease continues to be a global health problem despite the development of vaccines owing to their moderate efficacy, limited protection, and challenges in vaccine access and distribution. This is also attributed to the evolution and development of resistance by the parasite and the vector towards some of the commonly used anti-malarials, such as chloroquine, artemisinin, and artesunate, as well as the commonly known insecticide (DDT) [6–10]. Therefore, there is an urgent need to find new alternative strategies that can help in the fight against malaria [10–14]. This is imperative as failing to find a solution could lead to catastrophic complications, resulting in a global malarial epidemic outbreak [2, 15].

Throughout history, people, and traditional healers, especially in Africa, have used conventional medicinal plants in macerations, extracts, steam baths, and concoctions to treat and cure a broad spectrum of diseases and ailments, including malaria [16–19]. Thus, highlighting the inherent therapeutic potential of the phytochemicals harboured within these plants as a good source of lead compounds [16, 17]. This is evidenced by the discovery of artemisinin and quinine from *Artemisia annua* and *Cinchona officinalis* [20, 21]. Therefore, because of this, medicinal plants are now seen as an appealing strategy in the fight against malaria, making validating and exploring the medicinal plants suspected to have anti-malarial potential imperative [16, 22]. This study focused on the scientific validation of *Ziziphus mucronata* and *Xysmalobium undulutum*, medicinal plants used by herbal practitioners in South Africa to treat malaria. *Xysmalobium undulutum* is a robust geophytic plant belonging to the family *Apocynaceae* and is widely distributed across South Africa. This plant is traditionally used for various ailments such as diarrhoea, abdominal pain, and postpartum cramps [23]. Historically, the plant has a long-standing commercial history as extracts of its roots have been commercialized as Uzura and used for non-specific diarrhoea [23, 24]. The plant has also been shown to possess a wide range of biological activities including anti-inflammatory, central nervous system effects, and antioxidant activity [24, 25]. The roots of the plants are suspected to have anti-malarial activity [25]. Similarly, *Ziziphus mucronata* is a multi-purpose, spiny, medium-sized tree that falls under the family *Rhamnaceae* and is widely distributed in South Africa [26]. This plant is traditionally used to treat about 46 infections and diseases including sexually transmitted infections, skin infections, and chest infections [26, 27]. Moreover, it has also been shown to exhibit biological activity against several conditions, such as diabetes and inflammatory problems, and is also suspected of anti-malarial activity [27]. These activities are attributed to the plant’s production of various phytochemicals, including saponins, terpenoids, fatty acids, and sterols [27]. However, these plants lack scientific validation to confirm their safety and efficacy and identify malaria-specific active compounds [27, 28].

Another appealing avenue in the fight against malaria is studying and targeting some malarial proteins, especially those deemed prospective anti-malarial targets. PfHsp70-1 is one of the many proteins expressed by *P. falciparum* that are considered future anti-malarial drug targets [14, 29, 30]. This is because this ubiquitous canonical protein is believed to be implicated in the survival and virulence of the parasite through its ability to facilitate a myriad of functions (protein folding processes, prevention of protein aggregates, and formation of multimolecular complexes) [14, 29–31]. This protein is expressed throughout the parasite's blood stage, where it plays a significant role in maintaining proteostatis, making it important in the parasite's survival [14]. It is also implicated in the development of resistance by *P. falciparum,* making its inhibition using small molecular compounds important in the development of effective anti-malarials, as these have the potential to lead to an independent chaperone function arrest and the arrest of downstream cascade reactions caused by the protein's ability to interact with other co-chaperones [32, 33]. Therefore, this study also targeted the PfHsp70-1 using isolated compounds from the two medicinal plants under investigation.

## Methods

### Materials

Unless stated otherwise, all the chemical reagents and materials used in this study were purchased from Sigma-Aldrich (South Africa), Glenham (South Africa), Merck KGaA (Germany), ACE (South Africa), Merck (South Africa), Sigma (USA), Thermo Scientific (USA), Bio-Rad (South Africa). Instruments used include ChemiDock TM MPImaging System (Bio-Rad, South Africa), UV–Vis 1800 Spectrophotometer (Shimadzu, Japan), Allerga TM 25R Centrifuge (Beckman Coulter, USA), Eppendorf Centrifuge (Sigma-Aldrich, South Africa), Sonicator probe (Bandelin Sonoplus, Merck, South Africa), NanoDrop (Bio-Tek) and AvantiTM J-30I Centrifuge (Coulter, USA), BioTek Synergy HT Microplate Reader (BioTek), Buchi-Rotary Evaporator (MRC, SA), and Nuclear Magnetic Resonance Spectrometer (Thermo Scientific, USA).

### Plant collection and preparation

The plant materials (bark of *Z. mucronata* and root of *X. undulutum*) were collected from Rhobokfontein, Zululand District, KwaZulu-Natal, South Africa (27°23′ 32.57'' latitude/South, 31° 29′ 37.46'' longitude/East). These plants were then identified and transported to the University of Johannesburg, Auckland Park, South Africa, where they were prepared for extraction. Before extraction, the plants were washed, air-dried, and pulverized into a fine powder and stored at ambient temperature until further use.

### Extraction and isolation

The powdered material of each plant species underwent sequential extraction. Briefly, 1 kg of each plant species was macerated in hexane in a ratio of 1:10 (w/v) for three days with intermittent shaking. The extracts were then filtered in a Whatman paper No. 1, with the remaining residue further macerated using the same fresh hexane solvent. This was followed by filtration and mixing of the filtrates/extracts. The remaining residue was then air-dried, and the same protocol was followed for ethyl acetate, dichloromethane, and methanol. A Buchi-rotary evaporator was utilized under reduced pressure and low temperature (< 50 °C) to remove the solvents from the extracts. The crudes were then left to air dry under the fume hoods, with the percentage yield of each extract calculated using formula (i). Efforts at isolating the active compounds were directed at the DCM extract of *Z. mucronata* due to its high yield (4.25 g per 1000 g of plant extract ~ 0.43%) and its observed anti-malarial activity, which was achieved using Silica gel chromatography. Gradient elution using solvents of different ratios (HEX: ETA at 9:1,8:2,7:3,6:4) was utilized to elute the column. A 15 mL each of three hundred and two (302) were collected from the column and analysed using thin-layer chromatography (TLC) by spotting them onto the TLC plate and viewing the spots under a UV light at 245 nm. The TLC plate indicated that 88 and 96 of the fractions found at 8:2 and 7:2, respectively were pure, these were then grouped, air-dried and their percentage yield was measured and found to be (8:2, 135 mg per 4.25 g of extract ~ 3.16%—betulinic acid, and 7:3, 7.65 mg per 4.25 g of extract ~ 0.18%) and stored at 4ºC until further use.

$$\left( {\text{i}} \right){\text{ Plant percentage yield}}\, = \,{\text{crude extract}}/{\text{amount of starting material}}\, \times \,{1}00.$$

### Structural elucidation

After the isolation of the compounds, anti-malarial and cytotoxic activity assays were conducted. After that, the compounds were sent to the Chemistry Department at the University of Johannesburg, where they were structurally elucidated using spectroscopic techniques (1H/Proton NMR (Nuclear Magnetic Resonance), ^13^C/Carbon NMR, and Distortionless Enhancement by Polarization Transfer (DEPT)). The resulting spectral data were then analysed by Prof F.O Shode (Organic Chemist), who identified the compounds as betulinic acid and lupeol.

### In vitro anti-malarial activity assay

The H3D Research group at the University of Cape Town carried out the crude extracts and isolated compound anti-malarial activity. They were tested against the NF54 *P. falciparum* chloroquine-sensitive strain in duplicates. Briefly, continuous in vitro culture of the asexual blood stage of the parasite was maintained following a previously modified method described by Trager and Jensen [34]. In vitro*,* anti-malarial activity was investigated using the parasite lactate dehydrogenase activity assay, a technique described by Makler et al*.* [35]. The crude extracts and isolated compounds were first prepared in a stock solution of 10 mg/mL in 100% dimethyl sulfoxide (DMSO), with the samples that could not dissolve in DMSO treated as suspensions. The stock solutions were diluted to a desired starting concentration, with 1 µg/mL of chloroquine and artesunate used as reference drugs. A full dose response was then performed for all the crude extracts to find the concentration at which 50% (IC_50_—value) of the parasite growth was inhibited. The crude extracts and compounds' primary concentrations were tested at 20 µg/mL and successively diluted twice as much in the growth medium to produce measures with varying concentrations. The assay plate was then incubated at 37 °C for 72 h in a sealed gas chamber at 3% O2, 4% CO2, and 93% N2. The plate wells were gently resuspended, with 15 µL from each well transferred to a duplicate plate containing 100 µL Malstat reagent and 25 µL of Nitroblue tetrazolium solution. After that, the plate was allowed to develop for 20 min in the dark, with the absorbance values read at 620 nm in a spectrophotometer. The remainder of the parasite population, each concentration of the test samples, was then determined by comparing the absorbance values of each well to the absorbance of the untreated well.

### Cytotoxic activity assay

The cytotoxicity assay of the crude extracts and isolated compounds was carried out by the H3D Research Center at the University of Cape Town using the Chinese Hamster Ovary (CHO) cell line and 3-(4,5- dimethylthiazol-2-yl)-2,5-diphenyltetrazolium bromide (MTT) assay. MTT assay works by measuring the metabolic activity of cells as a function of cell viability, proliferation, and cytotoxicity by reducing MTT into a formazan salt using succinate-tetrazolium reductase found within cells [10]. The assay was done using a previously modified method described by Mosman [36]. Briefly, the crude extracts and isolated compounds were prepared to a 10 mg/mL stock solution using 100% DMSO, with extracts and compounds that could dissolve tested as suspensions. The crude extracts and isolated compounds were diluted to the desired starting working concentrations using a growth media. CHO cells were developed to semi-confluency in cell culture flasks and seeded to a density of 10^5^ per well using a 96-well plate. This was followed by a 24-h incubation period of the plate at 37℃ and 5% CO2. After that, varying concentrations (20 µg/mL to 16 ng/mL) of the crude extracts, compounds, and emetine as a positive control were added to the plate in triplicates and incubated at 37 °C for 48 h. After 48 h, the old medium was removed, and a new media and 5 mg/mL MTT were added to the PBS. The plate was incubated for 4 h, after which MTT and media were discarded. Then, 200 µL of DMSO was added to dissolve the formazan, and the plate was read at 540 nm using a spectrophotometer. The percentage cell viability of the cells was calculated in reference to the positive control using formula (ii). This was followed by plotting the percentage viability against the IC_50_ concentrations obtained using a non-linear dose–response fitting analysis using Dotmatics software.

$$\left( {{\text{ii}}} \right){\text{ Percentage viability}}\, = \,{\text{treated cells}}/{\text{control}}\, \times \,{1}00.$$

### Molecular docking

Due to the absence of a solved crystal structure of PfHsp70-1. A homology model of the protein was created using SWISS-MODEL (https://swissmodel.expasy.org/interactive). The protein sequence (Accession number: PF3D7_0818900) was retrieved from the National Centre of Biotechnology Information (NCBI) and submitted to SWISS-MODEL, where a template with high sequence similarity and Global Model Quality Estimation (GMQE) was selected to build the model. The resulting model was downloaded in a protein data bank (PDB) format. This format was then prepared for molecular docking using UCF Chimera Version 1.17.1 by removing water molecules, if any, and adding polar hydrogen bonds. The structures of the compounds (lupeol and betulinic acid) were downloaded from PubChem (https://pubchem.ncbi.nlm.nih.gov/) and deposited in the Molegro Molecular viewer, where they were protonated with partial charges. Energy minimization of the compounds was carried out using Avogadros General Amber Field Force (GAFF), with the compounds having the lowest possible potential energy saved in a mol.2 format. AutoDock Vina was then used to perform the compounds’ docking against the protein using default settings and site-directed docking. This was done by housing the protein's nucleotide-binding domain (NBD) under the following grid parameters: x = 40, y = 40, z = 40 dimensions, x = -5, y = 0.0, and z = -11 centers. Docked conformations of the receptor-ligand complexes were generated using a Lamarckian genetic algorithm approach in order of their docking scores. They were then viewed using UCSF Chimera and 2-dimensional protein–ligand complexes prepared using LigPlot.

### Recombinant protein expression of PfHsp70-1

The recombinant protein expression of PfHsp70-1 was achieved following previously described protocols with minor modifications [14, 37]. Chemically competent *Escherichia coli* BL21 (DE3) cells were transformed with pQE30-PfHsp70-1 plasmid DNA, followed by inoculation of the cells into 100 mL of 2X YT (1.6% tryptone, 1.0% yeast extract, 0.5% NaCl, pH 7.4) media supplemented with 100 µg/mL ampicillin and the incubation of the cells in a shaker incubator overnight at 165 rpm and 37 °C The resulting inoculum was sub-cultured in 900 mL of 2X YT media and placed back into the shaker incubator until the optical density was mid-log phase (OD600—0.6). Overexpression of PfHsp70-1 was induced using 1 mM isopropyl-β-D-1-thiogalactopyranoside (IPTG) at 25 °C Hourly samples were taken for 6 h and 24 h post-induction, followed by centrifugation and analysis using a 12% sodium dodecyl sulfate–polyacrylamide gel electrophoresis (SDS-PAGE). The remainder of the overnight culture (24 h post induction) was harvested using the High-speed Avanti JI30I centrifuge for 20 min at 10 000 Xg with the pellet resuspended in a Tris lysis buffer (100 mM Tris–HCl, pH 7.4, 300 mM NaCl, 10 mM imidazole, 1X lysis reagent, 1X protease inhibitor cocktail) and stored at − 80 °C.

### Purification of the recombinant PfHsp70-1

The cells suspended in lysis buffer and stored at − 80 °C were thawed on ice and mildly sonicated (4 × cycles of 30 s, 60% power) and 30 s incubation on ice between each cycle. After that, 0.1% w/v of poly(ethyleneimine) was added to the lysate to precipitate nucleic acids. The resulting lysate was centrifuged at 5 000 Xg for 30 min, following which the clear lysate was loaded and incubated for 4 h into an equilibrated HisPur nickel-charged nitrilotriacetic (Ni–NTA) column. After the incubation period, non-specific proteins were washed off from the column with wash buffer 1 (100 mM Tris–HCl, pH 7.4, 300 mM NaCl, 25 mM imidazole, 1X protease inhibitor cocktail) and twice with wash buffer 2 (100 mM Tris–HCl, pH 7.4, 300 mM NaCl, 50 mM imidazole, 1X protease inhibitor cocktail). The bound protein was eluted using elution buffer 1 (100 mM Tris–HCl, pH 7.4, 300 mM NaCl, 25.

mM imidazole, 1X protease inhibitor cocktail) and twice with elution buffer 2 (100 mM Tris– HCl, pH 7.4, 300 mM NaCl, 500 mM imidazole, 1X protease inhibitor cocktail). The collected fractions from the column were analysed in a 12% SDS-PAGE gel and visualized using a ChemiDock imaging system. The pure protein was then dialyzed extensively using a pre-soaked snakeskin tubing (MW 7000) in a dialysis buffer (10 mM Tris, 150 mM NaCl, 10% glycerol, 0.8 mM DTT) and aliquoted in 1.5 mL Eppendorf tubes and stored at − 20 °C.

### Investigation of the effect of betulinic acid and lupeol on the structural elements of PfHsp70-1 using UV–vis spectroscopy

Conformational and structural changes in the PfHsp70-1 by betulinic acid and lupeol were studied using UV–Vis spectroscopy. This was carried out by preparing 0.4 µM of PfHsp70-1 in 2 mL Eppendorf tubes and varying concentrations of the compounds (2 mM, 1 mM, 0.5 mM, and 0.25 mM). The reaction was then transferred into a 96-well plate in triplicate, followed by incubating the plate into the Synergy HT Microplate reader for an hour at 25 °C Conformational and structural changes were then monitored at a wavelength ranging from 200 to 400 nm with 20 nm increments.

### Investigation of the effect of betulinic acid and lupeol on the chaperone function of PfHsp70-1 using the malate dehydrogenase aggregation suppression assay

PfHsp70-1 has been demonstrated to prevent the heat-induced aggregation of malate dehydrogenase enzyme from the porcine heart [14]. Therefore, in this study, we sought to investigate the effect of betulinic acid and lupeol on the chaperone function of PfHsp70-1 following a previously described protocol with minor modifications [38]. Briefly, 0.4 µM of MDH was prepared in an assay buffer (50 mM Tris, 100 mM NaCl, pH 7.5) with and without varying concentrations of the compounds (2 mM, 1 mM, 0.5 mM, 0.25 mM). This was followed by incubating the samples in a heat block set at 51 °C for 10 min before adding PfHsp70-1. After adding the protein, the samples were transferred into a 96-well plate and incubated for 30 min. The aggregation of MDH was monitored as a function of light scattering at 360 nm, and the absorbance values were expressed as a function of % aggregation relative to MDH alone. For controls, 2 mM polymyxin B (PMB) and 1 mg/mL BSA were utilized.

## Results

### In vitro anti-malarial and cytotoxicity activity assays of the crude extracts

As seen in Table 1, all the plant extracts except the methanol extract of *Z. mucronata* and *X. undulutum* demonstrated considerable anti-malarial activity against the parasite. However, their level of anti-malarial potency varied. Notably, for *Z. mucronata*, the ETA (IC_50_ = 7.23 ± 1.41 µg/ml) and HEX (IC_50_ = 11.69 ± 3.89 µg/ml) extracts displayed moderate activity, while the DCM (IC50 = 5.49 ± 0.003 µg/ml) extract proved to be more potent. Similarly, for *X. undulutum*, the ETA (IC_50_ = 17.46 ± 0.0024 µg/ml) and DCM (IC_50_ = 19.27 ± 0.492 µg/ml) extracts exhibited moderate activity, while the HEX (IC_50_ = 4.98 ± 0.37 µg/ml) extract proved to exhibit exceptional anti-malarial potency. The cytotoxicity assay of the crude extracts indicated no adverse effects by *X. undulutum* as all the extracts CC_50_ were greater than 50 µg/ml. Conversely, in *Z. mucronata*, only the hexane plant extract had no considerable activity, with the ETA (CC_50_ = 10.96 µg/ml) and DCM (CC_50_ = 10.01 µg/ml) extracts of the plant demonstrated moderate activity against the CHO cell line.

**Table 1:** Anti-malarial and cytotoxicity activity of the selected South African medicinal plants and the isolated compounds against the *P. falciparum* chloroquine-sensitive strain (NF54) and Chinese Hamster ovary cell line (CHO)

Plant name	Solvent of extraction	Anti-malarial activity (IC_50_) (µg/ml)	Status of activity	Cytotoxicity (CC_50_) (µg/ml)	Status of cytotoxicity
*Z. mucronata*	Hexane	11.69 ± 3.84	Good	> 50	Minimal/no cytotoxicity
	Ethyl acetate	7.23 ± 1.41	Good	10.96	Moderate
	Dichloromethane	5.49 ± 0.03	Good	10.01	Moderate
	Methanol	> 50	Minimal/no anti-malarial activity	ND	ND
*X. undulutum*	Hexane	4.98 ± 0.37	Maximum	> 50	Minimal/no cytotoxicity
	Ethyl acetate	17.46 ± 0.024	Moderate	> 50	Minimal/no cytotoxicity
	Dichloromethane	19.27 ± 0.492	Moderate	> 50	Minimal/no cytotoxicity
	Methanol	> 50	Minimal/no anti-malarial activity	ND	ND

Sample	Elution solvent	Anti-malarial activity (IC_50_) (µg/ml)	Status of activity	Cytotoxicity (CC_50_) (µg/ml)	Status of cytotoxicity
Compound A/lupeol	7:3 (Ethyl acetate/hexane)	7.56 ± 2.03	Good	> 50	Minimal/no cytotoxicity
Compound B	8:2 (Ethyl acetate/hexane)	19.95 ± 1.53	Moderate	> 50	Minimal/no cytotoxicity
Chloroquine	Control	0.010 ± 0.001	Maximum	ND	Minimal/no cytotoxicity
Artesunate	Control	0.005 ± 0.001	Maximum	ND	Minimal/no cytotoxicity

^**^ND: Not determined, N/A: Not applicable, IC50: Half maximal inhibitory concentration, CC50: Half maximal cytotoxic concentration

### In vitro anti-malarial and cytotoxic activity assays and structural elucidation of the isolated compounds

**Fig. 1:**
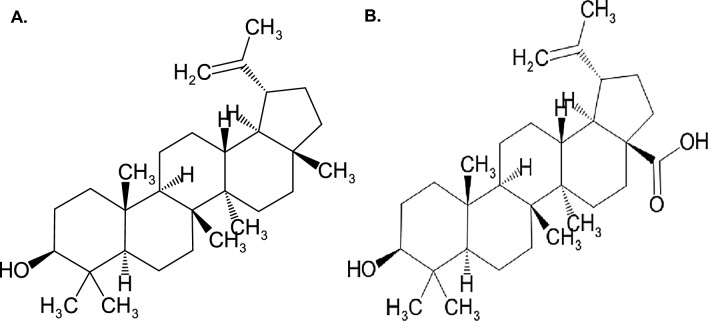
Chemical structures of the compounds isolated from the *Z. mucronate* dichloromethane extract, (1) Lupeol; (2) Betulinic acid

Isolation efforts to identify the compounds responsible for the observed anti-malarial activity of the crude extracts were directed towards the DCM extract of *Z. mucronata* due to its high percentage yield (0.427% per 1000 g of extract) despite it being moderately cytotoxic. As indicated in Table 1, the isolated compounds were found to induce anti-malarial activity (Compound A/lupeol = 7.56 ± 2.03 µg/ml; Compound B/Betulinic acid = 19.95 ± 1.53 µg/ml) against the blood stage parasite with no considerable cytotoxicity (CC50 > 50 µg/ml). Compound A proved to be 38% more potent than compound B. However, both the compounds were less potent when compared to the parent extract (DCM extract = 5.49 ± 0.03 µg/ml) as well as the positive controls: chloroquine (IC_50_ = 0.010 ± 0.001 µg/ml) and artesunate (IC_50_ = 0.005 µg/ml ± 0.001). Structural elucidation revealed that compound A was lupeol and compound B was betulinic acid Fig. 1.

### Molecular docking analysis of the interaction between lupeol and betulinic acid

**Fig. 2:**
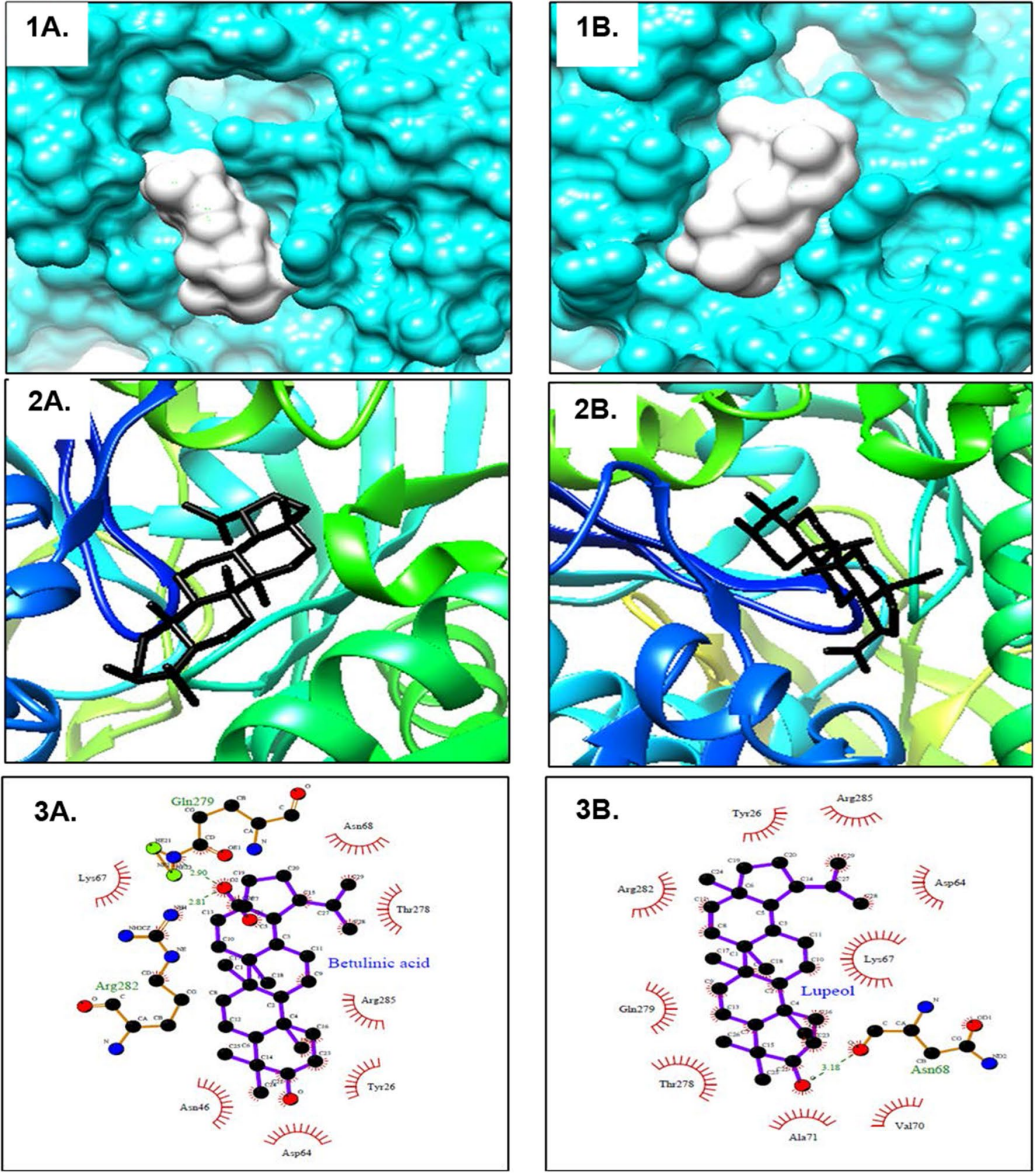
Schematic visualization of the protein–ligand bond interactions between PfHsp70-1 and the isolated compounds. Showing the 3-dimensional space-filling (1), surface and ribbon view (2), and the 2-dimensional protein–ligand interface (3) models of the docked conformations, poses, and binding modes of lupeol (**A**) and betulinic acid (**B**) in the nucleotide-binding domain (NBD) of PfHsp70-1. Lupeol (3A) creates a single hydrogen bond with ASN 68. At the same time, betulinic acid (3B) makes two hydrogen bonds with Gln 279 and Arg 289, with the rest of the interactions being hydrophobic interactions from both models

**Fig. 3:**
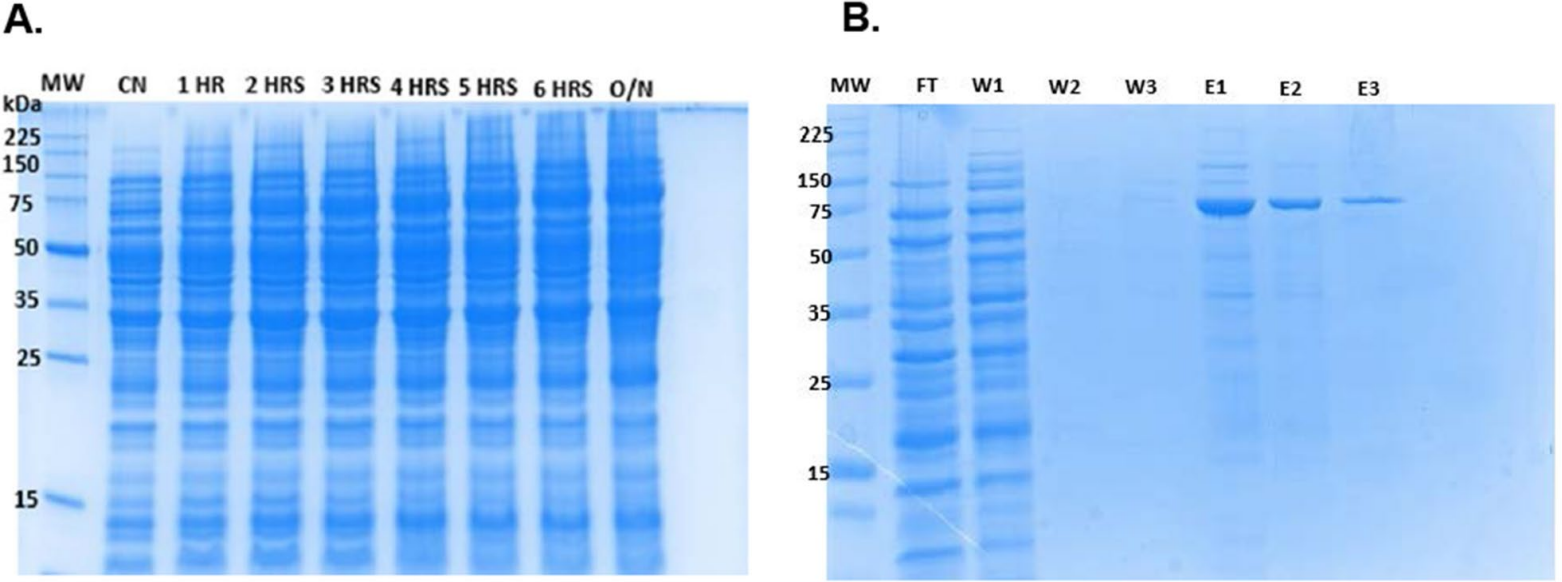
12% SDS-PAGE gel for the **A** expression and **B** purification of the recombinant PfHsp70-1 expressed in *E. coli* BL21 DE3 cells using 0.5 mM IPTG at 25 °C. Lane MW represents the molecular marker, CN: control samples/uninduced cells, lane 1-6 h: hourly samples taken post induction of protein expression, O/N: Overnight expression, FT: Flow-through, W1-W3: Washes, and E1-E3: Elutions

PfHsp70-1 is a critical role player that aids in the survival and development of resistance by the parasite [14, 32, 39]. Making its inhibition important in the fight against malaria [39]. Therefore, following the structural elucidation of the isolated compounds, the study investigated and assessed the potential binding and inhibition of PfHsp70-1 by these compounds. This was done using site-directed molecular docking, a computational technique widely used to fast-track drug discovery by predicting and modelling protein–ligand interactions [40]. As seen in Fig. 2 (1A-2A, 1B-2B), the space-filling (1) and surface and ribbon view (2) of the protein–ligand interactions indicated that lupeol (A) and betulinic acid (B) can dock into the nucleotide-binding domain (NBD) of PfHsp70-1 supported by the RMSD scores of 0.00 (Table 2), that suggest the formation of stable complexes between the protein and the ligands. Table 2 also reveals that lupeol has a strong binding affinity against the protein, shown by its significant binding score of − 6.8 kcal/mol. This aligns with Mishra and Day [41], who stated that a binding score below 5 kcal/mol indicates negligible binding. The two-dimensional interface done using LigPlot (Figs. 2, 3A) showed that the interactions between lupeol and the protein were governed by a single hydrogen formation with amino acid Asn68 (bond length: 3.18 Å), signifying a strong and stable interaction. Several hydrophobic interactions between the ligand and amino acids (Arg282, Gln279, Thr278, Ala171, Val70, Lys67, Asp68, and Arg285) were also notable, suggesting the stability and high-affinity binding of the ligand to the protein consistent with Tou et al*.* [42], who states that hydrogen bonds and hydrophobic interactions are critical molecular forces that govern docking stability. Betulinic acid also demonstrated strong binding affinity to the protein with a binding score of − 6.9 kcal/mol, characterized by forming two hydrogen bonds between amino acid Gln279 (2.90 Å) and Arg282 (2.81 Å). Like lupeol, several notable hydrophobic interactions between betulinic acid and the amino acids (Thr278, Asn68, Arg285, Tyr26, Asp64, and Lys67) were also observed, indicating the formation of stable and strong binding of betulinic acid to PfHsp70-1 (Figs. 2, 3B). These findings indicate the potential binding of these compounds in the nucleotide-binding domain of PfHsp70-1, suggesting the potential ability of the compounds to compete and inhibit the ATP/ADP substrates of PfHsp70-1, which could lead to the disruption of the catalytic ATPase activity of the protein and chaperone function.

**Table 2:** Molecular docking outputs illustrating the docking scores and the types of bond interactions between isolated compounds (betulinic acid and lupeol) and PfHsp70-1

Compound	RMSD (Å)	Docking score (Kcal/mol)	Hydrogen bonds	Hydrophobic interactions
Lupeol	0.00	− 6.9	Asn68	Arg282, Gln279, Thr278, Ala171, Val70, Lys67, Asp68, and Arg285
Betulinic acid	0.00	− 6.8	Arg282 and Gln279	Thr278, Asn68, Arg285, Tyr26, Asp64, and Lys67)

^**^RMSD: Root mean square deviation

### Recombinant expression and purification of PfHsp70-1

To validate the molecular docking outputs that suggested the binding of lupeol and betulinic against the PfHsp70-1. In vitro validation and assays were performed by expressing, purifying, and characterizing the protein in the presence of the compounds. As shown in Fig. 3A, the expression of the protein was successful and resolved at the expected theoretical size of 74 kDa, consistent with the findings by Salomane et al*.* [14]. The protein also proved to be expressed before the induction of protein expression, suggesting leaky expression, which usually occurs because of a weak and unregulated promoter (T5 promoter) [43]. Moreover, breakdown products of PfHsp70-1 were also observed at 35 kDa and 50 kDa, which is consistent with findings by Ramya and colleagues [44]. Following the successful expression of PfHsp70-1, the protein was purified using nickel affinity chromatography. As seen in Fig. 3B, the purification of the protein was successful, indicated by the presence of a band running at 74 kDa. However, some protein was lost in the flow through (Fig. 3B, lane FT) and wash 1 (Fig. 3B, lane W1). This could be due to the inability of the protein to bind to the column optimally and column saturation. Contaminants and non-specific proteins were also observed upon elution of PfHsp70-1 (Fig. 3B, lane E1), suggesting the potential presence of the *Escherichia coli* BL21 (DE3) histidine-rich proteins and the probable binding of the breakdown products of PfHsp70-1 [45]. The protein was, however, purified to homogeneity, as seen in lanes E2-E3 of Fig. 3B.

### UV-spectroscopic evaluation of the effect of lupeol and betulinic on PfHsp70-1

**Fig. 4:**
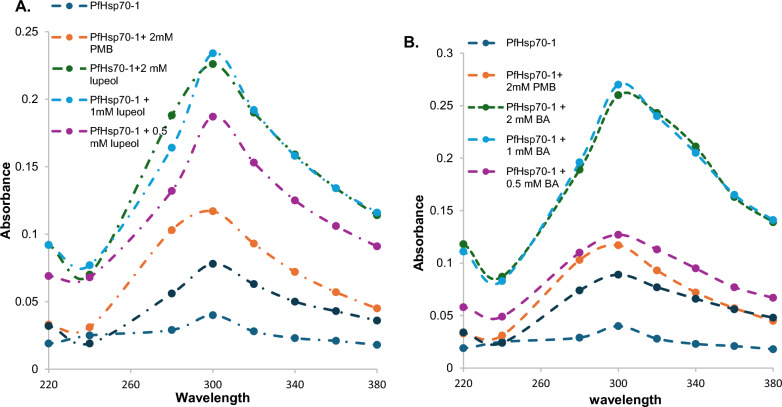
UV–vis spectrum used to study the effect of varying concentrations (0.25 mM- 2 mM) of **A** lupeol betulinic acid and **B** betulinic acid on the secondary structural elements of 0.4 µM of PfHsp70-1. The reaction was initiated and incubated for 2 h at 25 °C in a Synergy Plate reader. The secondary structural changes were monitored at a wavelength ranging from 200 to 400 nm with 20 nm increments. The key in the spectrums shows the concentrations of the compounds used

Several spectroscopic techniques have been developed to study protein–protein and protein–ligand interactions, one of which is UV–vis spectroscopy [45]. This technique monitors the protein secondary structural changes using three amino acids (Tyrosine, Phenylalanine, and Tryptophan) that are found in the core of a protein, where they drive the stability and folding state of a protein [45–47]. The results indicated in Figs. 4A and B indicate that the lupeol and betulinic acid induce structural changes in the PfHsp70-1. This is indicated by the increase in the absorbance values of the protein treated with the compounds relative to the untreated protein. The increase in absorbance values suggests that the compounds expose the hydrophobic amino acids and promote the unfolding state of the protein. The compounds also show that the disruption of the protein's secondary structural elements is dose-dependent. The compounds were also more effective than the known potential inhibitor of PfHsp70-, Polymyxin B, as shown by the increase in absorbance values at 0.5 mM for the compounds compared to 2 mM of the inhibitor (Figs. 4A and B). Betulinic acid also proved to be more effective against PfHsp70-1 when compared to lupeol. Contrary to the in vitro findings, lupeol is 38% more effective against the *P. falciparum* NF54 strain than betulinic acid.

### Evaluation of the effect of lupeol and betulinic acid on the chaperone function activity using the malate dehydrogenase aggregation suppression assay

**Fig. 5:**
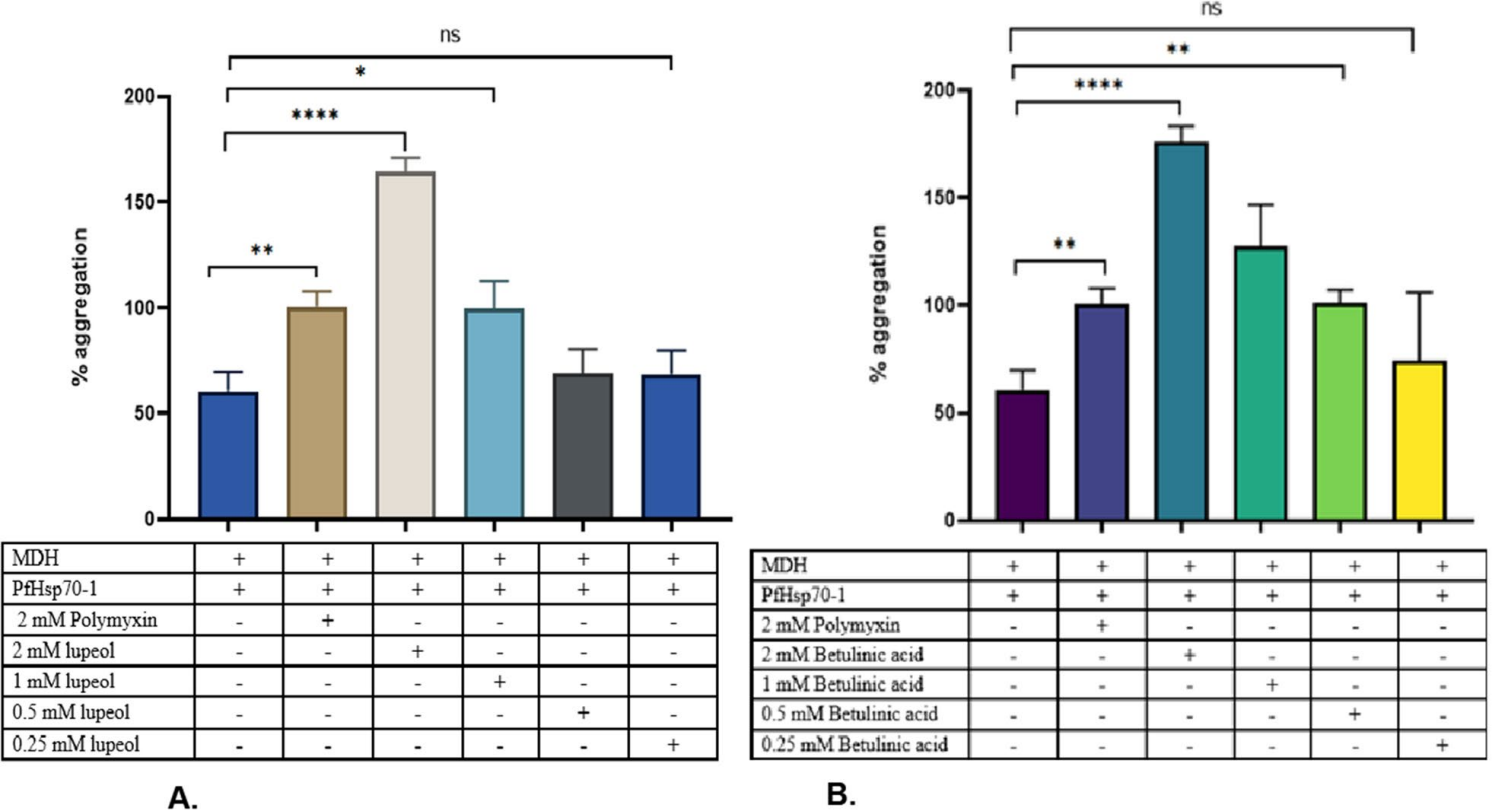
Effect of varying concentrations (0.25–2 mM) of (1) betulinic acid and (2) lupeol on the heat-induced malate dehydrogenase suppression activity of PfHsp70-1. This assay was monitored by exposing 1 µM of MDH to heat stress at 48 °C in the presence of 0.4 µM of PfHsp70-1 and varying concentrations of betulinic acid, lupeol, and polymyxin B (control). The heat-induced aggregation was monitored at 360 nm using an HT Synergy Plate reader and expressed as a function of percentage aggregation. The error bars show the average mean values generated from three replicates, and the statistically significant difference was conducted using one-way ANOVA (*p < 0.5, **p < 0.1, ***p < 0.001, and ****p < 0.0001)

PfHsp70-1 is a thermostable cytosolic protein that has been shown to suppress the heat-induced aggregation of some model proteins (malate dehydrogenase and luciferase) [14, 38]. This assay was therefore performed to investigate if the compounds can disrupt the PfHsp70-1 chaperone activity. However, compounds were tested on the protein and assessed for its chaperone activity on MDH. The results in the supplementary data (Fig. 6) indicate that MDH aggregated in the absence of PfHsp70-1 and that when PfHsp70-1 was added, the aggregation of MDH was reduced or reversed, shown by the decrease in the percentage aggregation when compared to MDH, proving that PfHsp70-1 is a heat stable and can suppress MDH aggregation [14, 38]. To confirm that the holdase function is PfHsp70-1 specific, BSA was used in place of PfHsp70-1, and as expected, the percentage of MDH aggregation increased. To test the potential effect of the compounds, the essay was repeated in the presence of varying concentrations of lupeol and betulinic acid. As seen in Fig. 5A, B, lupeol and betulinic acid were proven to inhibit the MDH suppression activity of the protein, as shown by the increase in percentage aggregation relative to MDH and the protein alone. This indicates that the compounds can disrupt the holdase chaperone function of PfHsp70-1. Moreover, like the UV–Vis results, the compounds (lupeol and betulinic acid) proved more effective when compared to PBM, and betulinic acid was more effective when compared to lupeol. However, betulinic acid was ineffective at 0.25 mM, and lupeol at 0.25 mM and 0.5 mM (Fig. 5A and B). While this data suggests the potential inhibition of the protein holdase function, further studies are warranted.

## Discussion

The alarming increase in the emergence of resistance by *P. falciparum* towards most of the widely used arials is a burdening hazard that has necessitated the acceleration of the need for new alternative approaches in the discovery and development of novel anti-malarials [10, 14]. Therefore, it has become critical to explore the phytochemistry of traditional medicinal plants, especially those suspected of having anti-malarial activity. This is important as some of the widely known anti-malarials, such as quinine and artemisinin, were plant-derived, highlighting plants’ potential to lead to the discovery of novel anti-malarials [10]. This study aimed to scientifically validate using *Z. mucronata* and *X. undulutum* in folk medicine to treat malaria.

The anti-malarial evaluation of crude extracts from these plants revealed intriguing patterns of anti-malarial activity when classified using the criterion described by Jonville et al*.* [48]. The methanol extracts from the two plants exhibited minimal anti-malarial activity as their IC_50_ values were greater than 50 µg/mL, signifying anti-malarial inactivity [39]. However, all the other extracts had varying degrees of anti-malarial potential. Notably, all the *Z. mucronata* plant extracts displayed promising anti-malarial activity as their IC_50_ values fell within the 6–15 µg/mL range. However, the DCM extract (IC_50_ = 5.49 ± 0.003 µg/mL) was more potent when compared to the HEX (IC_50_ = 11.69 ± 3.84 µg/mL) and ETA extract (IC_50_ = 7.23 µg/mL) [39]. In contrast, the *X. undulutum* HEX extract exhibited exceptional anti-malarial activity indicated by an IC_50_ value of 4.98 µg/mL, falling within the range displaying maximal anti-malarial activity (IC_50_ < 5 µg/mL) [39]. The ETA (IC_50_ = 17.46 ± 0.024 µg/mL) and DCM (IC_50_ = 19.72 ± 0.49 µg/mL) had moderate anti-malarial activity characterized by IC_50_ values ranging from 16 to 30 µg/mL.

Cytotoxic studies were performed to evaluate the cytotoxic nature of the crude extracts and substantiate their safety and therapeutic viability in folk medicine. As revealed in Table 1, all the extracts from *X. undulutum* showed no considerable cytotoxicity against the Chinese Hamster ovary (CHO) cell line. This is indicated by their respective CC_50_ values greater than 50 µg/mL, consistent with Garcia-Huertas et al*.* [34], who stipulated that an extract is potentially not toxic when its CC_50_ is greater than 50 µg/mL. In contrast, the hexane extract of *Z. mucronata* (CC_50_ > 50 µg/mL) showed no significant cytotoxicity, while the ETA (10.01 µg/mL) and DCM (10.96 µg/mL) extract indicated moderate activity [40]. Since these extracts underwent the process of air-drying, the cytotoxic nature of these extracts could not be attributed to the solvents of extractions, suggesting the potential presence of toxic metabolites or phytochemicals in the plant and their synergistic effect [17].

To facilitate the discovery of the compounds responsible for the observed anti-malarial potential observed in the crude extracts and the potential discovery of novel anti-malarials, isolation efforts were made towards the extracts exhibiting exceptional anti-malarial activity (HEX extract of *X. undulutum*). However, due to its low percentage yield, the isolation was performed from the DCM extract of *Z. mucronata* despite its moderate cytotoxicity owing to its high percentage yield (0.427% per 1000 g of extract). The rationale was that the compound responsible for the observed cytotoxic nature of the crude extract could be different from the compound responsible for the anti-malarial activity. As observed in Table 1, the rationale was proven true as the compounds (Compound A- lupeol and Compound B- betulinic acid) were able to induce anti-malarial activity against the asexual parasite of *P. falciparum* with no considerable cytotoxicity (CC_50_ > 50 µg/mL) [40]. Compound A (IC_50_ = 7.56 ± 2.03 µg/mL) also proved to be 38% more effective against the asexual blood stage parasite of *P. falciparum* when compared to compound B (IC_50_ = 19.5 ± 1.53 µg/mL). The compounds were also less effective than the mother extract (DCM extract, IC_50_ = 7.23 ± 1.41 µg/mL), suggesting that there could be synergistic activity between the isolated compounds and other metabolites in the plant, possible decomposition of the compounds during isolation and fractionation as well as removal of the protective matrix from the compounds [17, 49]. Moreover, the compounds were also less active when compared to the positive control (chloroquine, IC_50_ = 0.010 ± 0.001 and artesunate, IC_50_ = 0.005 ± 0.001 µg/mL), prompting the possible structural modification of the compounds to increase their anti-malarial potential [17, 50].

Following the anti-malarial and cytotoxic evaluation of the isolated compounds, the compounds were structurally elucidated (see supplementary data for spectral data, Figs. S1-6) and found to be lupeol and betulinic acid, which fall under a class of natural products known as triterpenes [51]. Therefore, the observed anti-malarial activity of these compounds was warranted as triterpenes are known to possess anti-malarial activity amongst other activities [17]. Lupeol, a pentacyclic triterpenoid, is known for its vast occurrence in various medically beneficial plants, shown by its presence in *Erythrina caffra* [49], *Ficus benjamina* [52], and *Holarrhena floribunda* [53]. Based on our current knowledge, lupeol has been isolated for the first time in *Z. mucronata*, highlighting the compound's variability and distribution in the plant kingdom and the study’s contribution to scientific literature. Lupeol has also been shown to possess anti-malarial activity against several *P. falciparum* strains including the *P. falciparum* chloroquine-sensitive 3D7 strain (IC_50_ = 45 μg/mL) and chloroquine-resistant FCR strain (IC_50_ = 41 μg/mL) [53]. It has also been shown to be effective against the *P. falciparum* (NF54) strain with an IC_50_ of 41.7 μg/mL, contrary to the findings of the study, which suggested that lupeol has an anti-malarial activity of 7.56 ± 2.03 μg/mL against the NF54 strain [49]. Betulinic acid is the other naturally occurring triterpenoid isolated in *Z. mucronata*. This compound has been shown to possess a broad spectrum of biological activities, including antibacterial, antioxidative, anti-inflammatory, and anti-malarial activity [54, 55]. This compound was previously isolated in *Z. mucronata* with butilin and ursolic acid [56]. However, these were said to possess antioxidant activity, proving the variability of the compound's biological activity [56]. A study by Steele et al*.* [57] also reported on the anti-malarial activity of the compound against *P. falciparum* CQ-resistant and sensitive strains with IC_50_ values of 19.6 μg/mL and 25.9 μg/mL, respectively. This data correlates with our findings, proving that betulinic acid has considerable anti-malarial activity. Although this is the case, Ziegler et al*.* [58] argue that the observed anti-malarial activity is not due to the toxic nature of the compound towards the parasite but due to chemical modelling and alterations of red blood cells by the compounds that lead to the inhibition of the parasite invasion and proliferation. This data, therefore, proves that lupeol and betulinic acid have the potential to be developed as prospective anti-malarials by enhancing their anti-malarial potency either through chemical modification or combination therapy.

## Conclusion

This study aimed to comprehensively analyse the anti-malarial and cytotoxic potential of *Z. mucronata* and *X. undulutum* to validate the use of medicinal plants in folk medicine. The findings of this study suggest that the crude extracts possess considerable anti-malarial potential, with some showing significant cytotoxic effects against normal cells. The findings also indicate that the anti-malarial potential, especially that of the dichloromethane extract of *Z. mucronata*, is attributed to the presence of 2 active compounds: betulinic acid and lupeol. When these compounds were studied for their inhibitory effect against PfHsp70-1, molecular docking revealed that these compounds tend to bind into the nucleotide-binding domain PfHsp70-1, suggesting their potential to inhibit the protein's ATPase activity. Validation of these computational outputs through the expression, purification, and characterization of the protein using UV–vis spectroscopy and the malate dehydrogenase activity assay indicated that both these compounds disrupt the protein secondary structural elements and the holdase chaperone activity of the protein by promoting the unfolding state of the protein. These results suggest that betulinic acid and lupeol could be potential drug candidates for developing anti-malarial drug chemotherapy and support the notion of considering medicinal plants as a biological reservoir for discovering and developing novel anti-malarial pharmaceutical agents.

### Supplementary Information

Supplementary Material 1.

## Data Availability

The data related to this article can be publicly available after the article is accepted. The authors confirm that the samples of the compounds are available from them.

## References

[1] Alebie G, Urga B, Worku A (2017). Systematic review on traditional medicinal plants used for the treatment of malaria in Ethiopia: trends and perspectives. Malar J.

[2] Sinha S, Medhi B, Sehgal R (2014). Challenges of drug resistant malaria. Parasite.

[3] Belachew EB (2018). Immune response and evasion mechanisms of *Plasmodium falciparum* parasites. J Immunol Res.

[4] WHO. Tackling emerging anti-malarial drug resistance in Africa. Geneva, World Health Organization, 2022. Available from: https://www.who.int/news/item/18-11-2022-tackling-emerging-antimalarial-drug-resistance-in-africa. Accessed 08–02–2023.

[5] Maharaj R, Seocharan I, Qwabe B, Mkhabela M, Kissoon S, Lakan V (2019). Decal epidemiology of malaria in KwaZulu-Natal, a province in South Africa, targeting elimination. Malar J.

[6] Percário S, Moreira DR, Gomes BA, Ferreira ME, Gonçalves AC, Laurindo PS (2012). Oxidative stress in malaria. Int J Mol Sci.

[7] Vasquez M, Zuniga M, Rodriguez A (2013). Oxidative stress and pathogenesis in malaria. Front Cell Infect Microbiol.

[8] Ntie-Kang F, Onguene PA, Lifongo LL, Ndom JC, Sippl W, Mbaze LM (2013). The potential of anti-malarial compounds derived from African medicinal plants, part II: a pharmacological evaluation of non-alkaloids and non-terpenoids. Malar J.

[9] Sato S (2021). *Plasmodium* - a brief introduction to the parasites causing human malaria and their basic biology. J Physiol Anthropol.

[10] Nyaba ZN, Murambiwa P, Opoku AR, Samson M, Shode FO, Simelane MBC (2018). Isolation, characterization, and biological evaluation of a potent anti-malarial drimane sesquiterpene from *Warburgia salutaris* stem bark. Malar J.

[11] Pwalia R, Joannides J, Iddrisu A, Addae C, Acquah-Baidoo D, Obuobi D, et al. High insecticide intensity of *Anopheles gambiae* (s.l) and low efficacy of pyrethroid LLINs in Accra, Ghana*.* Parasit Vectors. 2019;12:299. 10.1186/s13071-019-3556-y PMC6567633 31196222

[12] Tokponnon FT, Sissinto Y, Ogouyemi AH, Adeothy AA, Adechoubou A, Houansou T (2019). Implications of insecticide resistance for malaria vector control with long lasting insecticidal nets: evidence from health facility data from Benin. Malar J.

[13] Tajbakhsh E, Kwenti TE, Kheyri P, Nezaratizade S, Lindsay DS, Khamesipour F (2021). Antiplasmodial, anti-malarial activities and toxicity of African medicinal plants: a systematic review of literature. Malar J.

[14] Salomane N, Pooe O, Simelane MBC (2021). Iso-mukaadial acetate and ursolic acid acetate inhibit the chaperone activity of *Plasmodium falciparum* heat shock protein 70–1. Cell Stress Chaperone.

[15] Bawa S, Kumar S, Dabru S, Kumar R (2010). Structural modifications of quinoline- based anti-malarial agents: recent developments. J Pharm Biollied Sci.

[16] Saxena M, Saxena J, Nema R, Singh D, Gupta A (2013). Phytochemistry of medicinal plants. J Pharm Phytochem.

[17] Simelane MB, Shonhai A, Shode FO, Smith P, Singh M, Opoku AR (2013). Anti-plasmodial activity of some Zulu medicinal plants and of some triterpenes isolated from them. Molecules.

[18] Salmerón-Manzano E, Garrido-Cardenas JA, Manzano-Agugliaro F (2020). Worldwide research trends on medicinal plants. Int J Environ Res Public Health.

[19] Carvalho LP, Kreidenweiss A, Held J (2021). Drug repurposing: a review of old and new antibiotics for the treatment of malaria: identifying antibiotics with a fast onset of antiplasmodial action. Molecules.

[20] Saifi MA, Beg T, Harrath AH, Altayalan FSH, Quraishy SA (2013). Anti-malarial drugs: mode of action and status of resistance. Afr J Pharm Pharmacol.

[21] Pinheiro LCS, Feitosa LM, Gandi MO, Silveira FF, Boechat N (2019). The development of novel compounds against malaria: quinolines, triazolpyridines, pyrazolopyridines and pyrazolopyrimidines. Molecules.

[22] Habibi P, Shi Y, Grossi-de-Sa MF, Khan I (2022). Plants as natural source of natural and recombinant anti-malarial agents. Mol Biotechnol.

[23] Balogun FO, Tshabalala NT, Ashafa AOT. Antidiabetic medicinal plants used by the Basotho tribe of Eastern Free State: a review. J. Diabetes Res. 2016; 1–14. 10.1155/2016/4602820 PMC4942634 27437404

[24] Ghorbani M, Kaloga M, Frey HH, Mayer G, Eich E (1997). Phytochemical Reinvestigation of *Xysmalobium undulatum* Roots (Uzara) 1. Planta Med.

[25] SANBI. Xysamolbium undulutum (L.) Aiton f. var untulutum. Accessed: https://pza.sanbi.org/xysamolbium-undulutum [21–02–2023].

[26] Adebayo SA, Masoko P (2019). A review on *Ziziphus mucronata* Willd (Rhamnaceae) commonly used to treat infections and inflammation-related conditions in southern Africa. Academic J Med Plants.

[27] Mongalo NI, Mashele SS, Makhafola TJ. *Ziziphus mucronata* Willd. (Rhamnaceae): its botany, toxicity, phytochemistry, and pharmacological activities. Heliyon. 2020;6:e03708. 10.1016/j.heliyon.2020.e03708 PMC7170964 32322712

[28] Egamberdieva D, Mamedov N, Ovidi E, Tiezzi A, Craker L (2017). Phytochemical and pharmacological properties of medicinal plants from Uzbekistan: a review. J Medicinally Active Plants.

[29] Shonhai A, Boshoff A, Blatch GL (2007). The structural and functional diversity of Hsp70 proteins from *Plasmodium falciparum*. Protein Sci.

[30] Sharma D, Masison DC (2009). Hsp70 structure, function, regulation, and influence on yeast prions. Protein Peptide Lett.

[31] Makumire S, Zininga T, Vahokoski J, Kursula I, Shanghai A (2020). Biophysical analysis of *Plasmodium falciparum* Hsp70-Hsp90 organizing protein (PfHop) reveals a monomer that is characterized by folded segments connected by flexible linkers. PLoS ONE.

[32] Daniyan MO, Przyborski JM, Shonhai A (2019). Partners in mischief: functional networks of heat shock proteins of *Plasmodium falciparum* and their influence on parasite virulence. Biomolecules.

[33] Zininga T, Pooe OJ, Makhado PB, Ramatsui L, Prinsloo E, Achilonu I (2017). Polymyxin B inhibits the chaperone activity of *Plasmodium falciparum* Hsp70. Cell Stress Chaperones.

[34] TragerW, Jensen JB. Humanmalaria parasites in continuous culture. Science. 1976;193:673–5. 10.1126/science.781840 781840

[35] Makler MT, Ries JM, Williams JA, Bancroft JE, Piper RC, Gibbins BL (1993). Parasite lactate dehydrogenase as an assay for *Plasmodium falciparum* drug sensitivity. Am J Trop Med Hyg.

[36] Mosman T (1983). Rapid calometric assay for cellular growth and survival: application to proliferation and cytotoxicity assays. J Immunol Methods.

[37] Zininga T, Achilonu I, Hoppe H, Prinsloo E, Dirr HW, Shonhai A (2016). *Plasmodium falciparum* Hsp70-z, an Hsp110 homologue, exhibits independent chaperone activity and interacts with Hsp70-1 in a nucleotide-dependent fashion. Cell Stress Chaperones.

[38] Muthelo T, Mulaudzi V, Netshishivhe M, Dongola TH, Kok M, Makumire S (2022). Inhibition of *Plasmodium falciparum* Hsp70-Hop partnership by 2-phenylthynesulfonamide. Front Mol Biosci.

[39] Amusengeri A, Astl L, Lobb K, Verkhivker GM, Tastan BÖ (2019). Establishing computational approaches towards identifying malarial allosteric modulators: a case study of *Plasmodium falciparum* hsp70s. Int J Mol Sci.

[40] Surabhi S, Singh BK (2018). Computer-aided drug design: an overview. J Drug Deliv Ther.

[41] Mishra A, Dey S (2019). Molecular docking studies of a cyclic octapeptide-cyclosaplin from sandalwood. Biomolecules.

[42] Tou WI, Chen CYC (2012). In silico investigation of potential SRC kinase ligands from traditional Chinese medicine. PLoS ONE.

[43] Saida F, Uzan M, Odaert B, Bontems F. Expression of highly toxic genes in *E. coli*: special strategies and genetic tools. Curr Protein Pept Sci. 2006;7:47–56. 10.2174/138920306775474095 16472168

[44] Ramya TNC, Surolia N, Surolia A (2006). 15-Deoxyspergualin modulates *Plasmodium falc*iparum heat shock protein function. Biochem Biophys Res Commun.

[45] Opoku F, Govender PP, Pooe OJ, Simelane MB (2019). Evaluating iso-mukaadial acetate and ursolic acid acetate as *Plasmodium falciparum* hypoxanthine-guanine-xanthine phosphoribosyltransferase inhibitors. Biomolecules.

[46] Schmid XF. Biological macromolecules: UV-visible spectrophotometry. Encyclopedia Life Sci. 2001;1–4.

[47] Makwana KM, Mahalakshmi R (2015). Implications of aromatic–aromatic interactions: from protein structures to peptide models. Prot Sci.

[48] Jonville MC, Kodja H, Humeau L, Fournel J, De Mol P, Cao M (2008). Screening of medicinal plants from Reunion Island for anti-malarial and cytotoxic activity. J Ethnopharmacol.

[49] Chukwujekwu JC, de Kock CA, Smith PJ, Van Heerden FR, Van Staden J (2016). Antiplasmodial activity of compounds isolated from *Erythrina caffra*. S Afr J Botany.

[50] Egbubine CO, Adeyemi MM, Habila JD (2020). Isolation and characterization of betulinic acid from the stem bark of *Feretia canthioides* Hiern and its anti-malarial potential. Bull Ntl Res Cent.

[51] Garg A, Sharma R, Dey P, Kundu A, Kim HS, Bhakta T, Kumar A. Analysis of triterpenes and triterpenoids. Chapt. 11. In: Silva AS, Nabavi SF, Saeedi N, Nabavi SM (eds.). Recent Advances in Natural Products Analysis. 2020;393–426.

[52] Singh A, Mukhtar HM, Kaur H, Kaur L (2020). Investigation of antiplasmodial efficacy of lupeol and ursolic acid isolated from *Ficus benjamina* leaves extract. Nat Prod Res.

[53] Fotie J, Bohle DS, Leimanis ML, Georges E, Rukunga G, Nkengfack AE (2006). Lupeol long-chain fatty acid esters with anti-malarial activity from *Holarrhena floribunda*. J Nat Prod.

[54] Yogeeswari P, Sriram D (2005). Betulinic acid and its derivatives: a review on their biological properties. Curr Med Chem.

[55] Lou H, Li H, Zhang S, Lu H, Chen Q (2021). A review on preparation of betulinic acid and its biological activities. Molecules.

[56] Parkar F, Njue AW, Langat MK, Omolo JO (2021). Characterization of secondary metabolites from the berries of *Ziziphus mucronata* and their antioxidant properties. Nat Prod Res.

[57] Steele JCP, Warhurst DC, Kirby GC, Simmonds MSJ (1999). In vitro and in vivo evaluation of betulinic acid as an anti-malarial. Phytother Res.

[58] Ziegler HL, Franzyk H, Sairafianpour M, Tabatabai M, Tehrani MD, Bagherzdeh K (2004). Erythrocyte membrane modifying agents and the inhibition of *Plasmodium falciparum* growth: structure–activity relationships for betulinic acid analogues. Bioorg Med Chem.

