# Supplementary material for: Phytochemical evaluation of Ziziphus mucronata and Xysmalobium undulutum towards the discovery and development of anti-malarial drugs
Source: Malar J. 2024 May 11;23:141. doi: 10.1186/s12936-024-04976-1 (PMC11088772; doi:10.1186/s12936-024-04976-1)
Supplement: Supplementary file 1 — Supplementary Material 1. [file 12936_2024_4976_MOESM1_ESM.docx]

**Supplementary data**

**
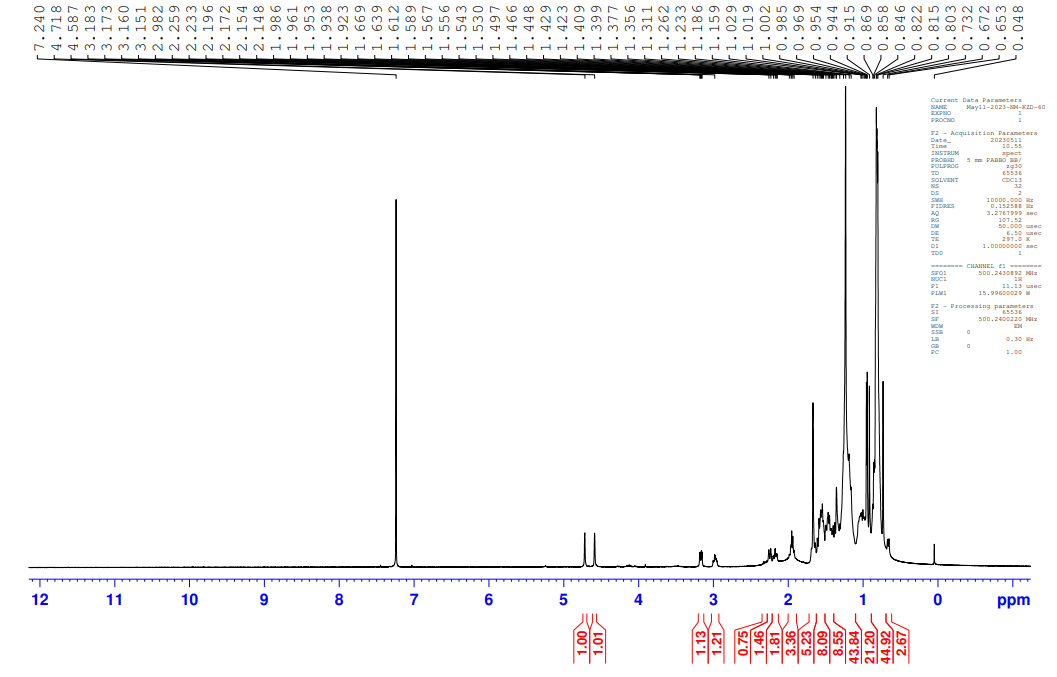
**

**Figure 1:** **^1^ H (proton) NMR spectrum for Betulinic acid,** showing chemical shifts between 0 ppm – 12 ppm.

**
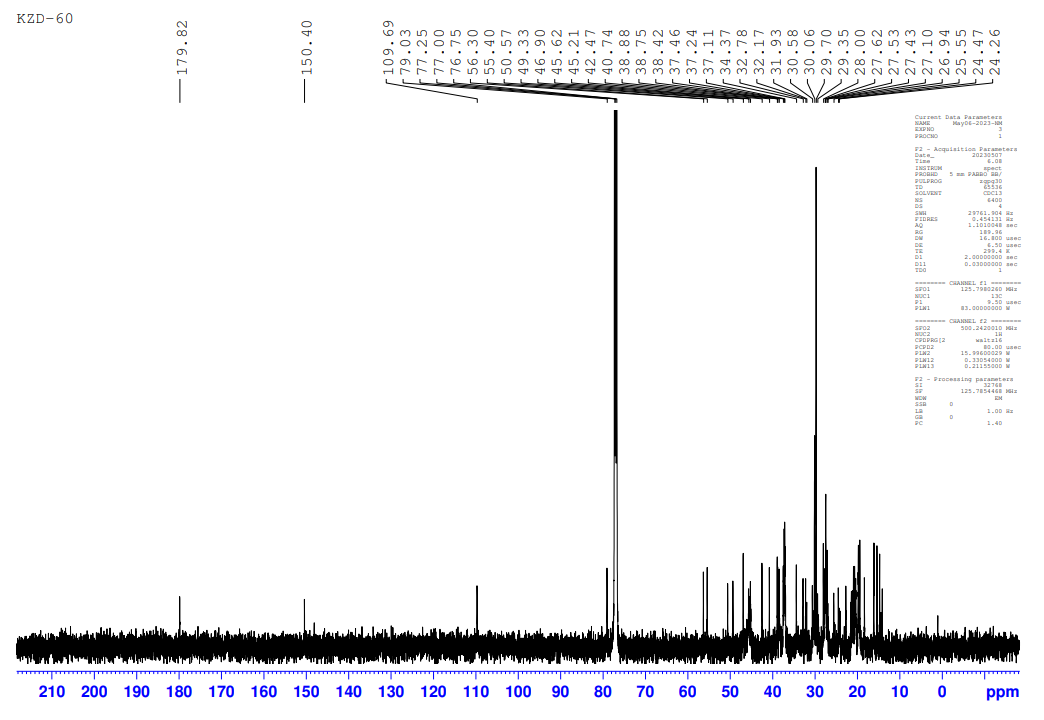
**

**Figure 2:** **^13^C (carbon) NMR spectrum for Betulinic acid,** showing chemical shifts between 0 ppm – 210 ppm.

**
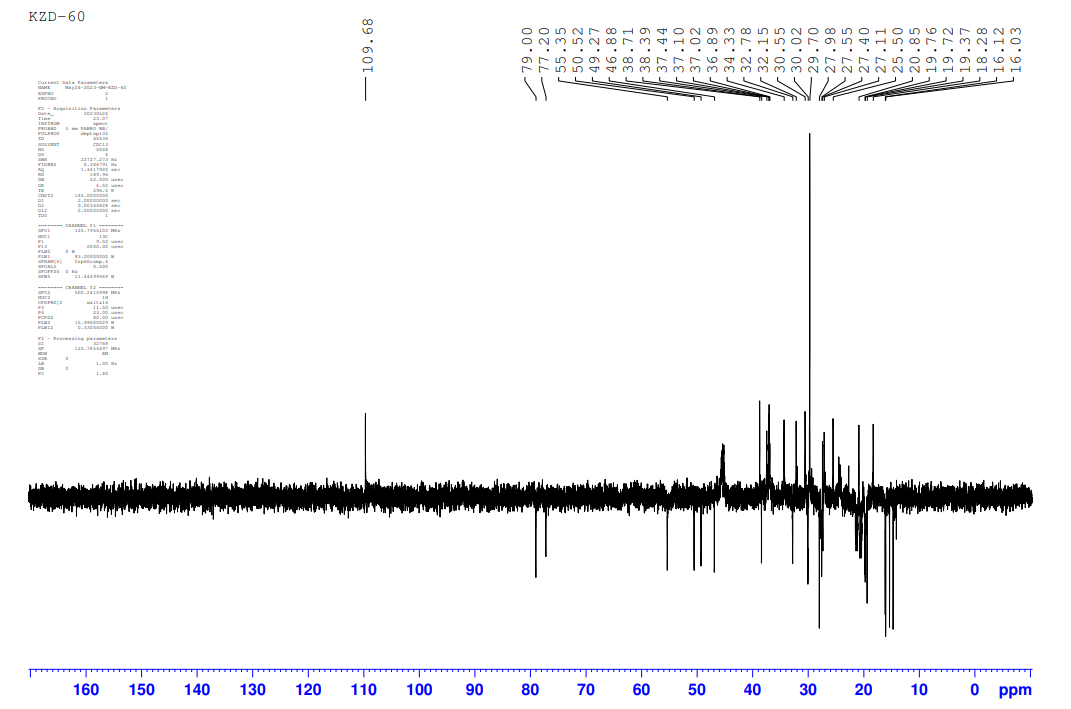
**

**Figure 3****: ^13^ C (Carbon) NMR-DEPT (Distortion less Enhancement by Polarization Transfer) spectrum for Betulinic acid.**

**
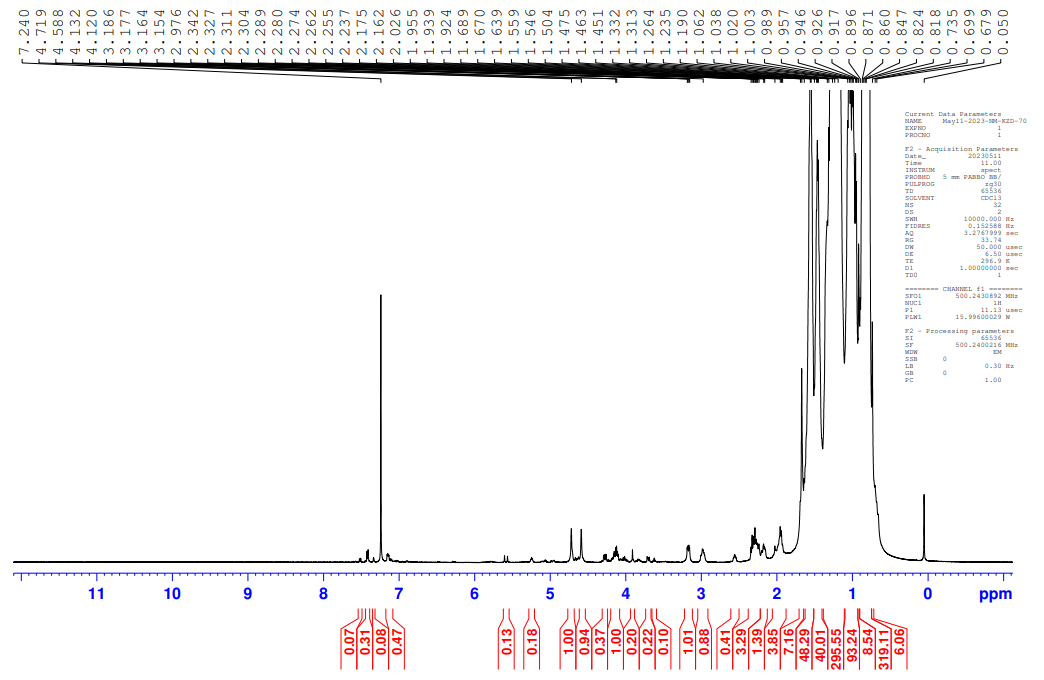
**

**Figure 4: ^1^ H (proton) NRM spectrum for Lupeol,** showing chemical shifts from 0 ppm – 11 ppm.

**
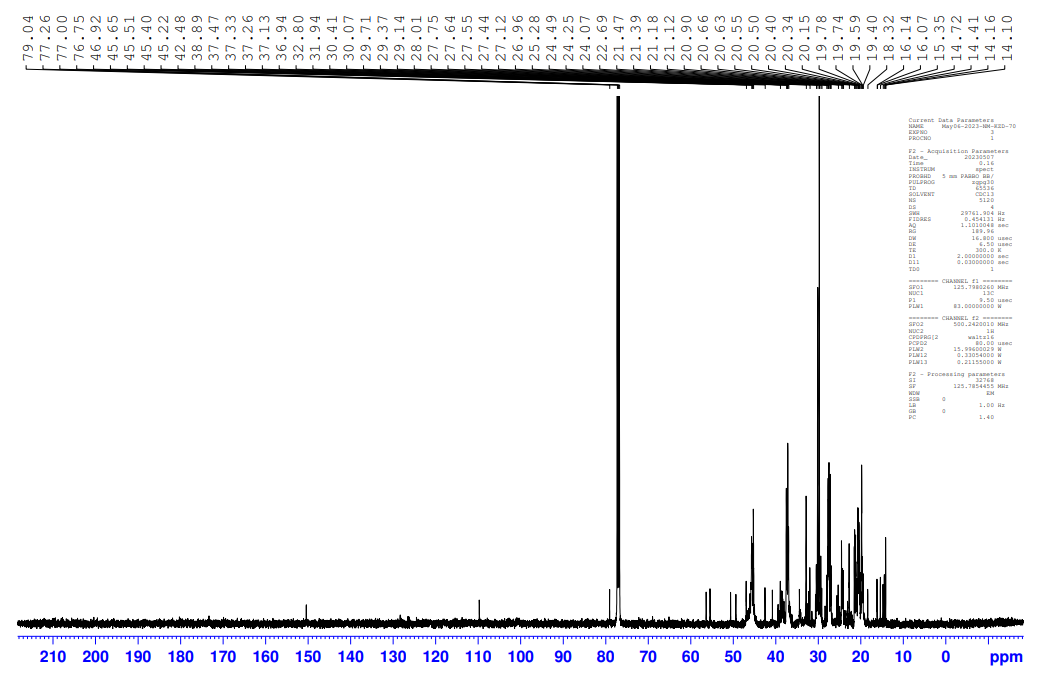
**

**Figure** **5: ^13^ C (carbon) NMR spectrum for Lupeol,** showing chemical shifts from 0 ppm- 210 ppm.

**
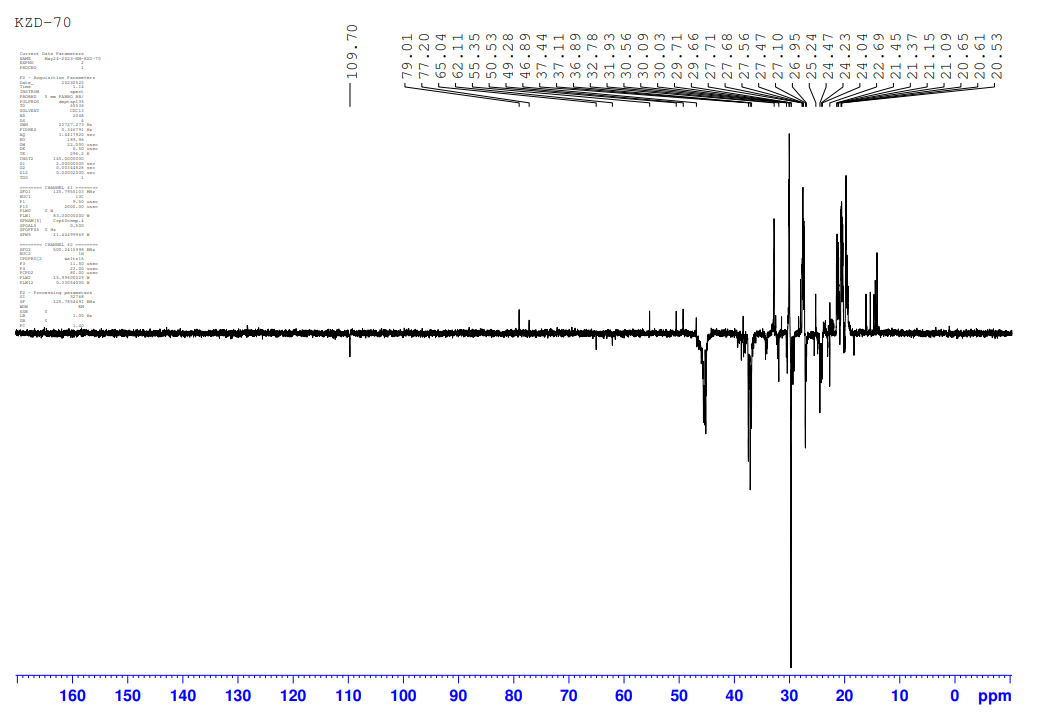
**

**Figure 6:** **^13^ C (Carbon) NMR-DEPT (Distortion less Enhancement by Polarization Transfer) spectrum for Lupeol**
